# Entangled Photon Pair Generation in the Telecom O‑Band from Nanowire Quantum Dots

**DOI:** 10.1021/acs.nanolett.5c01130

**Published:** 2025-06-02

**Authors:** Mohammed K. Alqedra, Chiao-Tzu Huang, Edith Yeung, Wen-Hao Chang, Sofiane Haffouz, Philip J. Poole, Dan Dalacu, Ali W. Elshaari, Val Zwiller

**Affiliations:** ‡ Department of Applied Physics, 7655 KTH Royal Institute of Technology, Roslagstullsbacken 21, 10691 Stockholm, Sweden; § Department of Electrophysics, 34914 National Yang Ming Chiao Tung University, Hsinchu 30010, Taiwan; ⊥ Research Center for Critical Issues, Academia Sinica, Tainan 711010, Taiwan; ∥ National Research Council of Canada, Ottawa, Ontario K1A 0R6. Canada; ¶ University of Ottawa, Ottawa, Ontario K1N 6N5, Canada

**Keywords:** nanowire quantum dots, entangled photons, telecom wavelength, single-photon sources, quantum-state tomography

## Abstract

Entangled photon pairs at telecom wavelengths are essential for quantum communication, distributed computing, and quantum-enhanced sensing. The telecom O-band offers low chromatic dispersion and fiber loss, which is ideal for long-distance networks. Site-controlled nanowire quantum dots have emerged as a promising platform for generating single and entangled photons, offering high extraction efficiency and scalability. However, their operation has largely been restricted to the visible and first near-infrared (NIR-I) windows. Here, we demonstrate a bright source of entangled photon pairs in the telecom O-band based on site-controlled nanowire quantum dots. We measure a fine-structure splitting of 4.6 μeV, confirming suitability for high-fidelity polarization entanglement. Quantum-state tomography of the biexciton–exciton cascade reveals a maximum fidelity of 85.8 ± 1.1% to the Φ^+^ Bell state and a maximum concurrence of 75.1 ± 2.1%. This work establishes nanowire quantum dots as viable entangled photon sources at telecom, advancing scalable quantum technologies for fiber-based networks.

## Graphical abstract

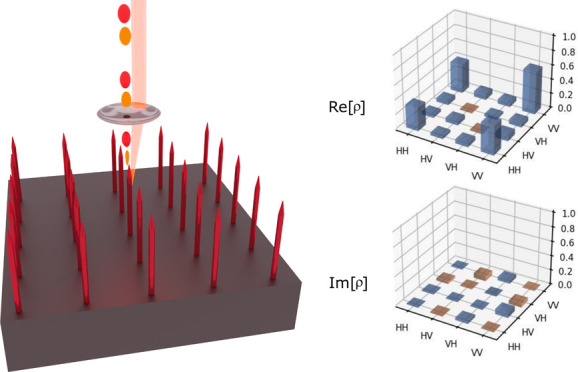

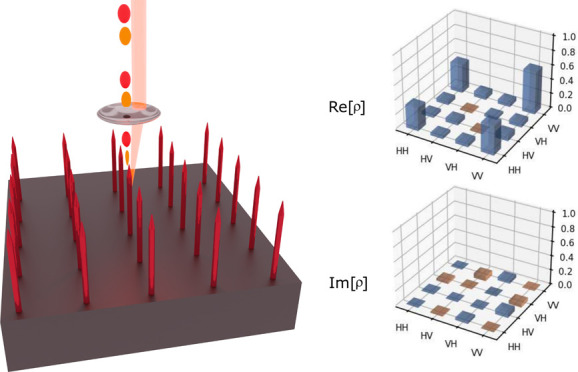

Entanglement is a key resource for various quantum technologies, including distributed quantum computing,^1^ quantum communication,^2,3^ and distributed quantum sensing.^4^ In quantum communication, entanglement enables protocols that can securely transmit information across long distances. When implemented effectively, it can extend the range of transmission by leveraging quantum repeaters and entanglement swapping techniques.^5,6^ For long-distance quantum communication over optical fibers, the telecom O-band (1260 to 1360 nm) is especially advantageous due to its near-zero chromatic dispersion and low transmission losses in standard fiber networks, making it suitable for quantum networks that aim to achieve high fidelity over large distances. Crucially, achieving these goals requires a reliable on-demand source of entangled photons capable of generating photon pairs deterministically and with high efficiency.^7–9^ Such sources overcome the limitations of probabilistic emitters, ensuring synchronized operation with quantum repeaters and maximizing the scalability of the quantum networks.

Photon-pair sources based on spontaneous parametric down-conversion (SPDC) have long served as the workhorse for demonstrations of fiber- and satellite-based quantum communication.^10,11^ However, as probabilistic sources, SPDC-based systems emit entangled photon pairs at random intervals, which limits their scalability and suitability for certain applications. Specifically, the random nature of SPDC emission complicates synchronization and hinders efficient operation with quantum repeaters, which require deterministic, on-demand photon generation for effective entanglement swapping over long distances. This lack of control also affects the overall system efficiency, often requiring complex multiplexing schemes to partially mitigate the inherent randomness.^12,13^

Epitaxially grown, self-assembled quantum dots (QDs) have shown great potential as sources of on-demand entangled photons, achieving high levels of brightness, purity, and indistinguishability.^14–16^ These QDs can be engineered to emit in specific telecom bands, aligning well with the low-loss transmission windows of optical fibers, which is crucial for quantum communication applications over long distances.^17–20^ However, despite these advantages, self-assembled quantum dots using lattice-mismatched semiconductors are randomly distributed, which complicates their localization and integration into scalable photonic devices. In contrast, site-controlled nanowire-based QDs present additional advantages for practical applications.^21–24^ The nanowire structure not only enables precise spatial control of the quantum dot’s position but also serves as a waveguide, enhancing the quantum dot’s brightness by efficiently directing emitted photons into the optical mode of the nanowire. This increased brightness improves the collection efficiency into optical fibers,^25^ making site-controlled, nanowire-based QDs an attractive option for practical quantum communication systems operating in the telecom O-band. In addition, site-controlled nanowire QDs offer a viable route for scalable integration of quantum dot emitters in advanced on-chip photonic structures.^26–35^

In this work, we demonstrate entangled photon-pair generation from site-controlled nanowire-based QDs in the telecom O-band. We perform a comprehensive spectroscopic characterization of the nanowire quantum dot and identify a biexciton–exciton (XX–X) cascade. We perform quantum-state tomography to characterize the entangled state generated by the QDs and calculate the fidelity and concurrence from the reconstructed density matrix. Our findings underscore the potential of nanowire-based QDs as bright, deterministic sources of telecom entangled photons for scalable and efficient quantum applications.

The quality of the entangled photon pairs generated by the XX–X cascade is strongly influenced by fine-structure splitting (FSS) of the quantum dot. This splitting arises from asymmetries in the quantum dot’s confinement potential. Such asymmetries can be caused by shape anisotropy or strain-induced distortions, which break the degeneracy of the bright exciton states. As a result, the exciton splits into two linearly polarized components with slightly different energies, leading to a time-dependent phase evolution in the polarization-entangled photon pair. The time-evolved entangled state emitted by a quantum dot with a FSS of Δ*E*_FSS_ can be written as

1 $$|\psi \left(t\right)\rangle =\frac{1}{\sqrt{2}}\left(|H_{XX}H_{X}\rangle +e^{i\Delta E_{\mathrm{FSS}}t/\hbar }|V_{XX}V_{X}\rangle \right)$$

where *ℏ* is the reduced Planck constant.

With fast detection systems, such as superconducting nanowire single-photon detectors (SNSPDs) used in the present work, it is possible to resolve the time-evolved entangled state introduced by the FSS. Although the emitted entangled state evolves over time due to the FSS, its fidelity does not decay; rather, the photons remain entangled, albeit in a time-dependent state. Thus, by utilizing a time-resolved scheme, all emitted photons can be utilized for certain quantum applications such as quantum key distribution (QKD), where the time evolution of the state is exploited rather than avoided.^36^

In addition, techniques to actively mitigate the effects of FSS further expand the usability of quantum-dot-based sources. Strain tuning has been previously used to control the FSS in bulk quantum dots by symmetrizing the confinement potential.^20^ However, applying strain effectively to nanowire quantum dots is more complex due to their distinct geometry, which limits efficient stress transfer to the embedded QD core. Although methods such as static piezoelectric actuation,^33,37^ deposition of stressor layers,^38^ dynamic modulation via surface acoustic waves,^31^ and gas condensation techniques^39^ have demonstrated emission wavelength tuning in nanowire quantum dots, their effectiveness in precisely controlling or eliminating FSS is still uncertain. Recent studies specifically indicate minimal influence on the FSS when employing these methods primarily for wavelength tuning.^39^ Thus, further dedicated studies are essential for systematically exploring strain-based FSS control in nanowire quantum dots. Alternatively, postemission processing schemes can be used to correct or compensate for the effects of FSS after photon emission.^40,41^ These methods, which involve active optical state manipulation or specific tomographic protocols, can ensure suitability for high-fidelity quantum applications even with nonzero FSS at the source.

## Method

The InAsP/InP nanowire quantum dots are grown from the bottom up via chemical beam epitaxy using a selective-area vapor–liquid–solid (SA-VLS) growth technique.^42^ First, an InP core with a diameter of ∼20 nm is grown. The process is briefly interrupted to grow a dot-in-a-rod structure consisting of a single InAs_0.68_P_0.32_ quantum dot of thickness ∼3 nm grown within an InAs_0.5_P_0.5_ nanowire rod of thickness ∼20 nm. Second, the core is cladded with an InP shell to produce a photonic waveguide with a base diameter of 310 nm tapered to 20 nm over the 12 μm length of the waveguide.^43,44^ A scanning electron microscopy (SEM) image of a section of the nanowire array is shown in Figure 1a, highlighting the uniformity and spatial arrangement of the nanowires. A higher-magnification SEM image of a single nanowire is shown in Figure 1b, revealing its tapered geometry. The schematic alongside the zoomed-in image provides a conceptual representation of the nanowire structure.

**Figure 1:**
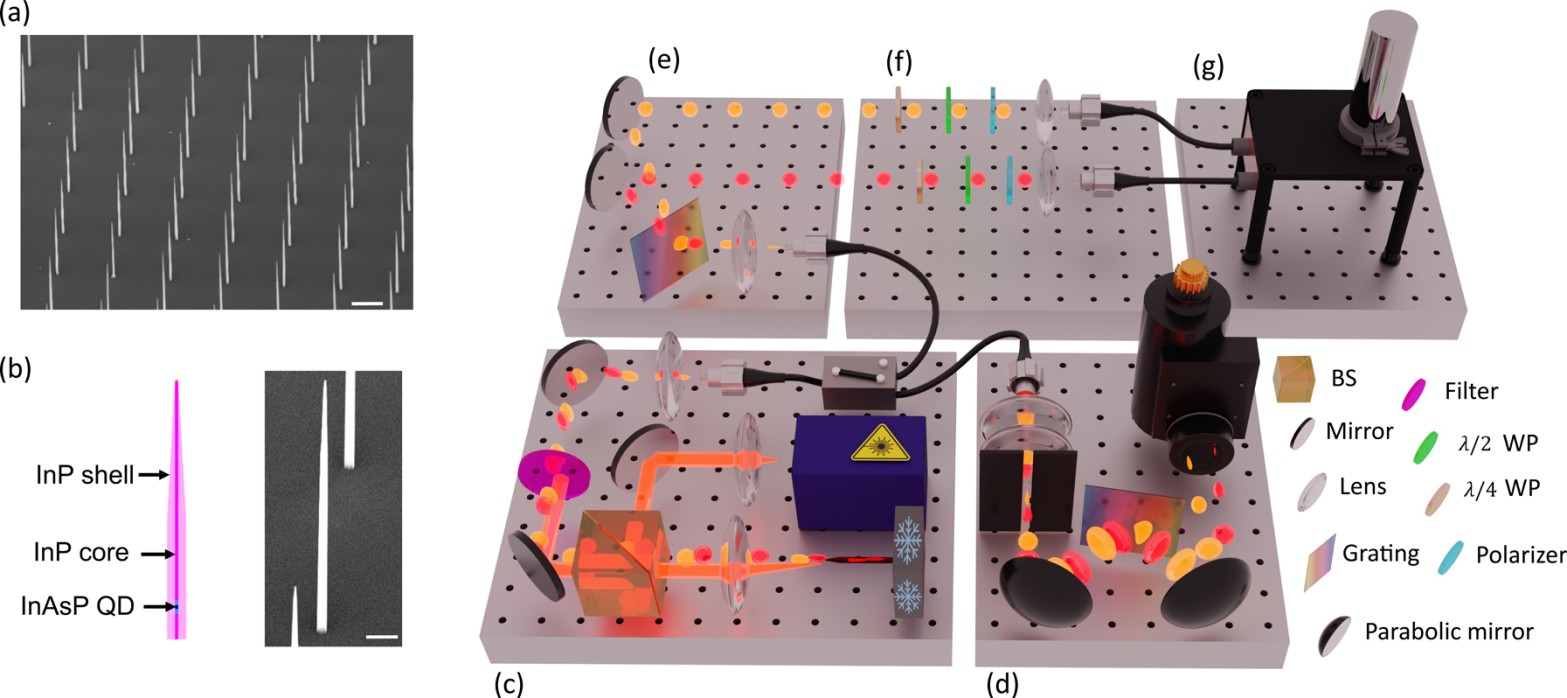
SEM image of the nanowires and schematic of the experimental setup used for QD characterization and quantum-state tomography. (a) SEM image of part of the nanowires array with (b) a higher-magnification image of a single nanowire and a schematic showing its structure. The scale bars are 4 and 1 μm, respectively. (c) QD excitation by a confocal setup. A power-stabilized 80 MHz laser is used to excite the QD into the p shell. The emission is collected by an objective with an NA of 0.8 and coupled into a single-mode fiber. (d) InGaAs spectrometer for spectral characterization of telecom emission. (e) TG for filtering the exciton and biexciton. (f) Quantum-state tomography setup. (g) SNSPDs.

The experimental setup, illustrated in Figure 1, is designed to excite the quantum dots and collect the emission through a confocal microscopy setup (Figure 1c) before directing the collected emission to different characterization modules for further characterization. An 80 MHz picosecond-pulsed laser source passes through a 10:90 beam splitter (BS). A total of 10% of the excitation light is directed toward the QD sample, while the remaining 90% is directed toward a photodiode used to provide feedback to an electronic variable optical attenuator for power stabilization. The QDs are housed within an Attodry 2100 cryostat operating at a temperature of 1.6 K. The sample scanning is achieved using cryogenic nanopositioners. An objective lens with 0.8 NA focuses the laser onto the QD and collects the emitted photons.

The emitted light from the QD passes through a 1250 nm long-pass filter to reject the excitation light and ensure that only the QD emission is collected into a single-mode SMF-28 fiber. The filtered light is then directed either to a spectrometer equipped with a liquid-nitrogen-cooled InGaAs diode detector for photoluminescence (PL) measurements (Figure 1d) or to a transmission-grating (TG) setup (Figure 1e) to spectrally filter X and XX.

The autocorrelation measurements were performed using a 50:50 fiber splitter to split the spectrally filtered emission into two paths, with each path directed to an SNSPD. The detected events from both SNSPDs are then time-stamped and digitized using a quTAG time-to-digital converter, enabling precise measurement of the arrival time correlations between the photons.

For entanglement measurements, the filtered XX and X photons are each directed into a polarization analysis setup consisting of a quarter-wave plate, a half-wave plate, and a polarizer (Figure 1f). The wave plates are mounted on motorized rotational stages, enabling precise control over the polarization basis. Full quantum-state tomography is performed by measuring a total of 36 projection bases to reconstruct the density matrix and accurately evaluate the entanglement fidelity and concurrence of the entangled photon pairs.

## Results

We first measured the PL properties of the nanowire quantum dot to characterize the excitonic states under above-band excitation at a 793 nm wavelength. The PL spectrum is shown in Figure 2a. From the spectrum, we can resolve emission from the s shell and the p shell. The inset shows the QD emission when excited into the p shell at 1190 nm, with the three highlighted peaks corresponding to the exciton (X), biexciton (XX), and charged exciton (T) states. These peaks are spectrally well-resolved, allowing for the clear identification and spectral filtering of each emission line for further characterization. The emission spectrum under p-shell excitation reveals additional weak peaks alongside the X, XX, and T lines. Based on the clean spectral signature observed under above-band excitation (see Figure 1 in Supporting Information S1) and the deterministic single-dot incorporation achieved via the SA-VLS growth method,^21,42,43^ we attribute these features to other excitonic states of the same quantum dot rather than emission from multiple dots.

**Figure 2:**
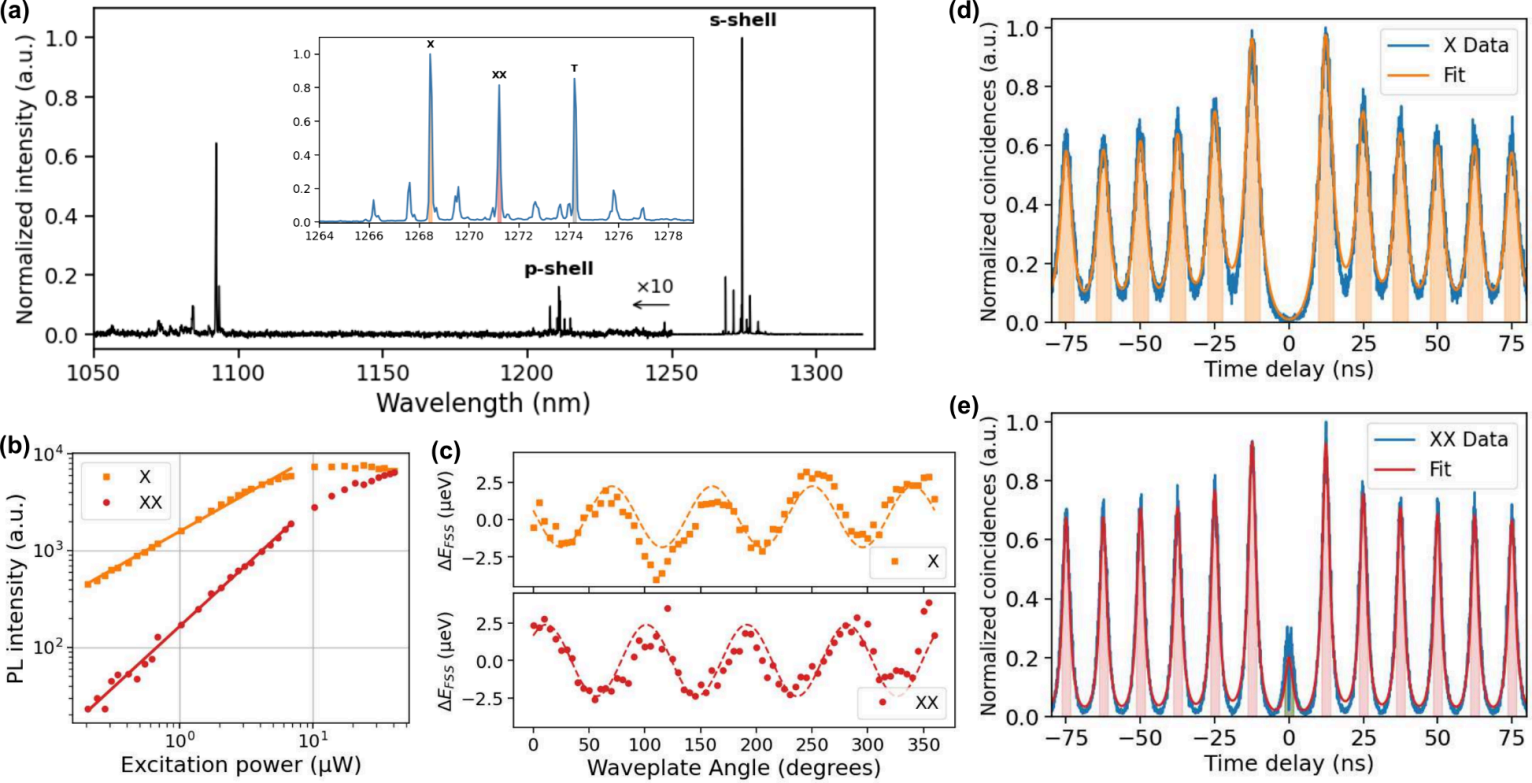
Spectroscopic characterization of the quantum dot emission. (a) QD emission spectrum measured with an InGaAs spectrometer when exciting above band at 793 nm. The inset shows the three most prominent peaks, when exciting into the p shell, corresponding to the exciton X (orange), biexciton XX (red), and trion T (gray). (b) Power-dependent measurement of the X and XX peaks. The exciton shows a sublinear dependence before it saturates around 10 μW, while for the biexciton, we observe a close-to-linear dependence. (c) Polarization-dependent measurement for both X (orange) and XX (red) to determine the FSS. The average splitting extracted from the sinusoidal fit (dashed line) is 4.6 μeV. (d and e) Measurements of the autocorrelation function *g* ^2^(τ) for X and XX, respectively. The solid line is the summed Lorentzian fit of the peaks, with the shaded region indicating the FWHM of each peak.

It should be noted that the wavelength used for p-shell excitation is slightly blue-shifted compared to the p-shell emission observed around 1210 nm when exciting below the band gap, as depicted in Figure 2a. When the excitation was tuned to 1210 nm, no emission was observed. This suggests that direct excitation to the p shell around 1190 nm wavelength is inefficient, likely due to weak dipole coupling or symmetry-related selection rules. Instead, the blue-shifted excitation at 1190 nm efficiently couples to higher-energy states within the p-shell manifold, which then relax nonradiatively to populate the lower-energy p-shell states, producing the observed emission.^45^ This observation underscores the significant role of phonon interactions in the excitation and relaxation dynamics of p-shell excitons, which should be investigated further.

Figure 2b shows the power dependence of these peaks, where the maximum intensities of the X and XX lines are plotted against the excitation power. The exciton exhibits a sublinear dependence with a slope of 0.78 and saturates at *P*_exc_ = 10 μW. The biexciton, on the other hand, displays a closer-to-linear dependence with a slope of 1.27. Both observed dependencies are below what is often reported in the literature for X, which should be linear, and for XX, which should be quadratic.^46^ The reason we observed these power relations could be explained by the existence of other competitive radiative decay paths formed by the charged exciton and charged biexciton. A more detailed explanation of this is presented in the Discussion section.

We characterized the FSS of the quantum dot by placing a motorized waveplate followed by a polarizing BS in front of the spectrometer. The waveplate angle was rotated in a full cycle with steps of 5°, recording the PL spectrum at each step. As the waveplate rotated, the polarization component of the X and XX emissions passing through the polarizing BS varies. This allows us to resolve the polarization-dependent energy shifts of the X and XX peaks, revealing the FSS. The observed splitting, indicative of asymmetries in the quantum dot confinement potential, is a critical parameter that influences the entanglement quality of the emitted photon pairs. Figure 2c shows the measured FSS oscillation of the X (orange) and XX (red) peaks as the waveplate rotates. The oscillation was fitted to a sinusoidal function (dashed lines), and the FSS extracted from the fitting was 4.6 μeV.

To assess the purity of the exciton and biexciton, we measured the autocorrelation function *g* ^2^(τ), as shown in parts d and e of Figure 2 for X and XX, respectively. This was performed with pulsed excitation of the p shell at 1190 nm. The zero-time-delay autocorrelation function, *g* ^2^(0), was determined by dividing the coincidence counts within a time window Δ, centered at zero delay, by the average of the summed coincidence counts within the same time window centered at each of the side peaks. The width of the time window, Δ, was obtained by fitting each side peak to a Lorentzian function and using the average full width at half-maximum (FWHM) from these fits. The obtained *g* ^2^(0) value for X was 0.024, indicating high single-photon purity. In contrast, the biexciton (XX) exhibited lower purity, with a *g* ^2^(0) value of 0.38. This higher *g* ^2^(0) for XX likely arises from a recapturing process under saturation excitation, as is evident by the narrow antibunching dip at zero delay. A more detailed discussion of this process can be found in Supporting Information S3. Additionally, bunching at longer time scales is observed in both autocorrelation traces, indicating blinking behavior under p-shell excitation. A quantitative analysis yields characteristic on/off time scales on the order of tens of nanoseconds for both X and XX. Details of the blinking model and fitting procedure are provided in Supporting Information S4.

Quantum-state tomography was performed by measuring 36 polarization projections. The analysis utilized the maximum likelihood estimation algorithm in ref 47, which was modified to reconstruct the density matrixes at each time bin across the cascade. Before measurement on the quantum dot, a calibration measurement was conducted using a classical V-polarized laser. The reconstructed state achieved a fidelity of 99.8% to the ideal V state, confirming the accuracy of the tomography setup. Details of this calibration measurement are provided in Supporting Information S6. Figure 3a displays the time-resolved cascade for the VV and HV polarization projections, revealing oscillations in the polarization state attributed to the FSS. The oscillation period is 890 ps, corresponding to an FSS of 4.65 μeV, in close agreement with the value extracted from the polarization-dependent measurement in Figure 2c. Systematic polarization rotations and birefringence introduced by optical elements preceding the tomography setup—including the cryostat window, collection optics, and initial collection fiber—can alter the photons’ polarization states. To correct this static polarization transformation, a virtual waveplate rotation with θ = 0.521 rad and ϕ = −1.284 rad was applied to the raw density matrixes at all time bins.^19,47^ The fidelity to the ideal Bell state Φ^+^, calculated from the corrected matrixes, reached a maximum of 85.8 ± 1.1% at the time bin highlighted in Figure 3b,c, where the concurrence peaked at 75.1 ± 2.1%, as shown in Figure 3c. The real and imaginary components of the reconstructed density matrix at the highlighted time bin are shown in parts d and e of Figure 3, respectively, exhibiting the characteristic features of a highly entangled state, with the real part prominently featuring the outer diagonal terms and the imaginary part exhibiting negligible contributions.

**Figure 3:**
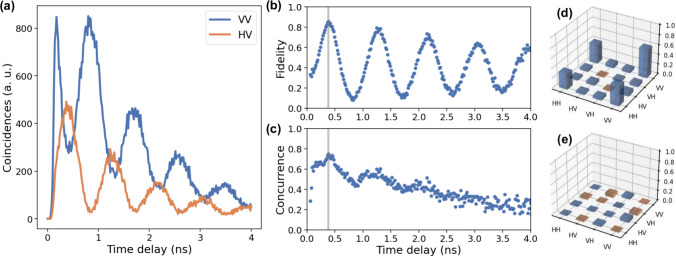
Quantum-state tomography of the telecom O-band entangled photons. (a) Coincidence measurement of the VV and HV basis. (b) Time-evolved fidelity to the Φ^+^ state. The oscillation is due to the FSS of the quantum dot. A maximum fidelity of 85.8 ± 1.1% is reached at the time bin highlighted in gray. (c) Reconstructed concurrence at different time bins across the cascade. A maximum concurrence of 75.1 ± 2.1% is reached at the same highlighted time bin. (d and e) Real and imaginary parts of the reconstructed density matrix at the highlighted time bin.

The measured fidelity could be further improved by utilizing resonant excitation methods, such as two-photon resonant excitation or phonon-assisted excitation, instead of the p-shell excitation used here. Such resonant approaches have been demonstrated to enable more coherent population of the biexciton state and reduce exciton–biexciton timing jitter, which would potentially lead to higher fidelity photon pairs.^24^

## Discussion

In the investigated nanowire system, different excitation schemes result in different power dependencies. Under p-shell excitation, the power series for X and XX exhibits significantly smaller slopes than expected, as shown in Figure 2b. For comparison, we investigated the power dependence of the PL under above-band-gap excitation. Instead of displaying the expected quadratic power relation for the biexciton emission, a closer to quadratic dependence is observed for another emission peak, which is attributed to the charged biexciton.^48^

This suggests that there are other radiative paths in the excitonic system that compete with the primary cascaded emission process. Because our nanowires have a negative background doping, it is more likely to form a charged biexciton than a neutral biexciton. A power dependence closer to a quadratic relation is more readily observed from the charged biexciton state. Under p-shell excitation, where carriers are predominately generated around the QD, more emission peaks are observed compared to above-band-gap excitation. This further indicates that these alternative radiative pathways significantly influence the carrier dynamics in the nanowire system. A more detailed discussion of this measurement can be found in Supporting Information S1 and S2.

Another noteworthy result is the strong recapturing effect observed in the autocorrelation measurement of the biexciton, which is absent in the exciton. This indicates that in a carrier-rich environment, under saturated excitation power, the QD tends to recapture another electron–hole pair to form the biexciton state after emitting a biexciton photon.^49^ When the central antibunching dip was fitted with a single-exponential decay, the recapturing time constant for forming a biexciton state was determined to be approximately 536ps (Supporting Information S3).

We also evaluated the source efficiency, which was estimated to be 12.5% at the first lens under pulsed p-shell excitation (Supporting Information S5). This value reflects the high brightness achievable with our nanowire platform. We note that self-assembled quantum dots coupled to high-*Q* microcavities have recently demonstrated system efficiencies as high as 70%,^50^ representing the current state-of-the-art in single-photon-source brightness. A comprehensive comparison of the growth methods and performance characteristics of self-assembled and nanowire quantum dots can be found in ref 51.

## Conclusion

To conclude, we have demonstrated polarization entangled photon-pair generation in the telecom O-band using a nanowire quantum dot. To the best of our knowledge, this represents the first measurement of telecom wavelength entanglement from nanowire quantum dots. The high brightness and low FSS of the dot allowed us to explore and characterize the entanglement properties of the photon pairs. By performing quantum-state tomography, we obtained a maximum fidelity of 85.8% and a concurrence of 75.1%, demonstrating a high degree of entanglement in the biexciton–exciton cascaded emission. These results were achieved by using p-shell excitation, and even higher levels of entanglement could potentially be reached by incorporating resonant excitation techniques. These results highlight the potential of nanowire quantum dots as bright, deterministic sources of entangled photons for scalable quantum communication and computing applications at telecom wavelengths, providing a foundation for their integration into advanced photonic devices and long-distance quantum networks.

## Supplementary Material



## Notes

Details on spectral characterization including additional spectra and lifetimes (S1), power dependence analysis under above-band-gap excitation (S2), biexciton purity analysis and recapturing mechanism (S3), blinking analysis (S4), source brightness and setup efficiency calculations (S5), tomography setup calibration (S6), and raw cross-correlation data for 16 projection measurements (S7) (PDF)
