# Supplementary material for: Entangled Photon Pair Generation in the Telecom O‑Band from Nanowire Quantum Dots
Source: Nano Lett. 2025 Jun 2;25(26):10321–7. doi: 10.1021/acs.nanolett.5c01130 (PMC12232380; doi:10.1021/acs.nanolett.5c01130)
Supplement: Supplementary file 1 [file nl5c01130_si_001.pdf]

Supporting Information: Entangled photon pair generation in the telecom O-band from nanowire quantum dots

Mohammed K. Alqedra,[*,1,†] Chiao-Tzu Huang,[2,3,†] Edith Yeung,[4,5] Wen-Hao Chang,[2,3] Sofiane Haffouz,[4] Philip J. Poole,[4] Dan Dalacu,[4,5] Ali W. Elshaari,[1] and Val Zwiller[*,1]

[1] *Department of Applied Physics, KTH Royal Institute of Technology, Roslagstullsbacken 21, 10691 Stockholm, Sweden*

[2] *Department of Electrophysics, National Yang Ming Chiao Tung University, Hsinchu 30010, Taiwan*

[3] *Research Center for Critical Issues, Academia Sinica, Tainan 711010, Taiwan*

[4] *National Research Council of Canada, Ottawa, Ontario, Canada, K1A 0R6*

[5] *University of Ottawa, Ottawa, Ontario, Canada, K1N 6N5*

[†] *Equal contribution*

* E-mail: alqedra@kth.se; zwiller@kth.se

S1. Spectral characterization of the quantum dot

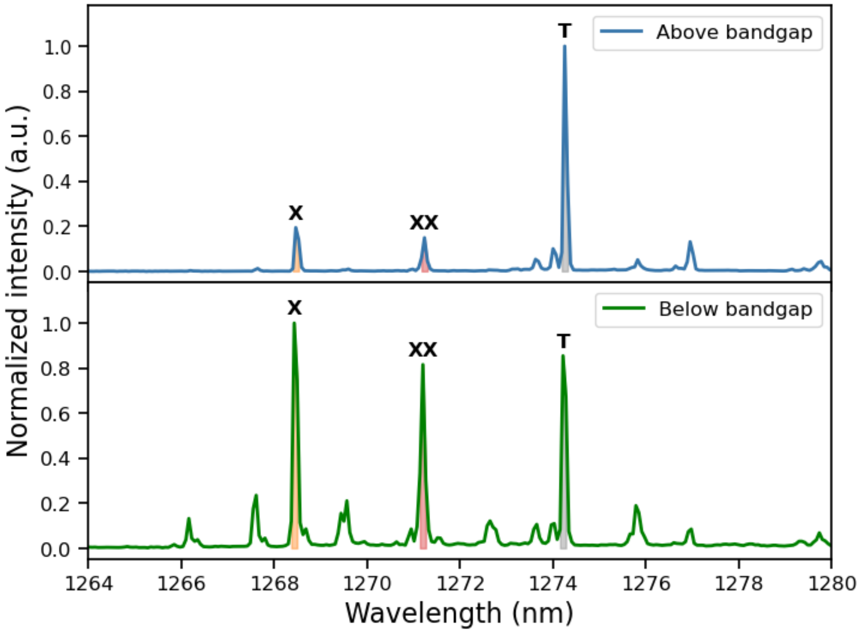

Figure 1: **Quantum dot emission at different excitation wavelengths.** The upper plot is for aboveband exciation at 793 nm. The lower plot is for belowband exciation at 881 nm wavelength

Here, we provide a detailed examination of the nanowire quantum dot emission under varying excitation conditions. Figure 1 illustrates the emission spectrum when exciting the quantum dot above the bandgap at 793 nm, and below the bandgap at 881 nm. We observe a strong suppression of the charged exciton state and a significant enhancement of the neutral exciton states when exciting below the band as compared to aboveband.

This difference arises from the distinct carrier generation and capture mechanisms in aboveband and belowband excitation. Aboveband excitation generates free carriers in the host material, which then relax into the quantum dot through scattering or phonon-assisted processes. This often results in an imbalance between electron and hole capture rates, increasing the likelihood of forming charged exciton states due to excess free carriers. In contrast, belowband excitation at 881 nm directly injects carriers into localized states near the quantum dot, bypassing the host material and minimizing the generation of excess free

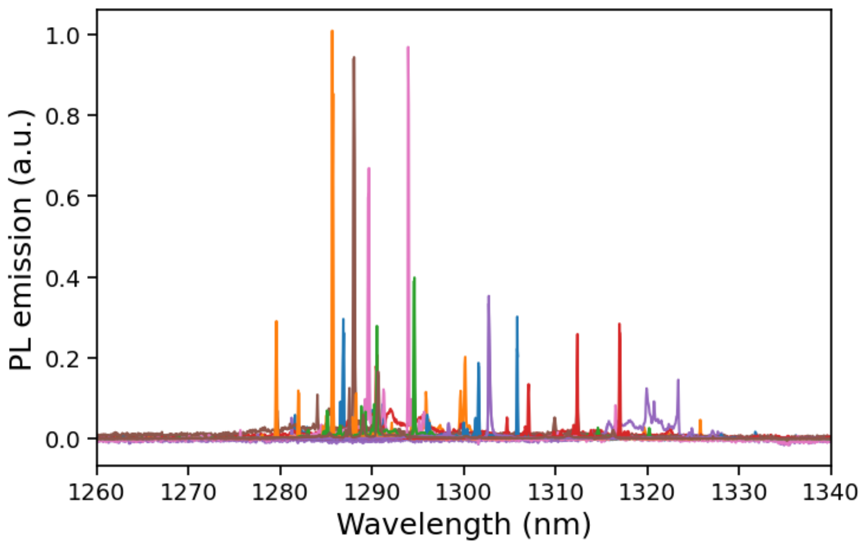

Figure 2: **Photoluminescence spectra of 12 randomly selected dots from the same sample under aboveband excitation.** Different nanowires are represented in different colors.

carriers. A similar observation is made with p-shell excitation, where carriers are excited directly into higher quantum dot energy levels before relaxing into the ground state. Both belowband and p-shell excitation provide controlled carrier injection, which suppresses the formation of charged excitons and enhances the emission of neutral exciton states. These observations confirm that excitation schemes targeting quantum dot states more directly minimize the generation of excess free carriers in the host material, suppressing unintended recombination processes and promoting the selective emission of neutral exciton states.

To evaluate the spectral properties and uniformity of the nanowire quantum dots, we measured the photoluminescence spectra of 12 randomly selected dots from the same sample when excited above the band. As shown in Figure 2, each spectrum, represented by a different color, corresponds to an individual nanowire quantum dot. All measured quantum dots, exhibit emission within the telecom O-band, indicating a high degree of spectral consistency across the sample. This confirms that the quantum dot investigated in the main text is representative of the broader distribution of emitters.

The radiative lifetime of the X and XX when excited to the p-shell is shown in Figure 3,

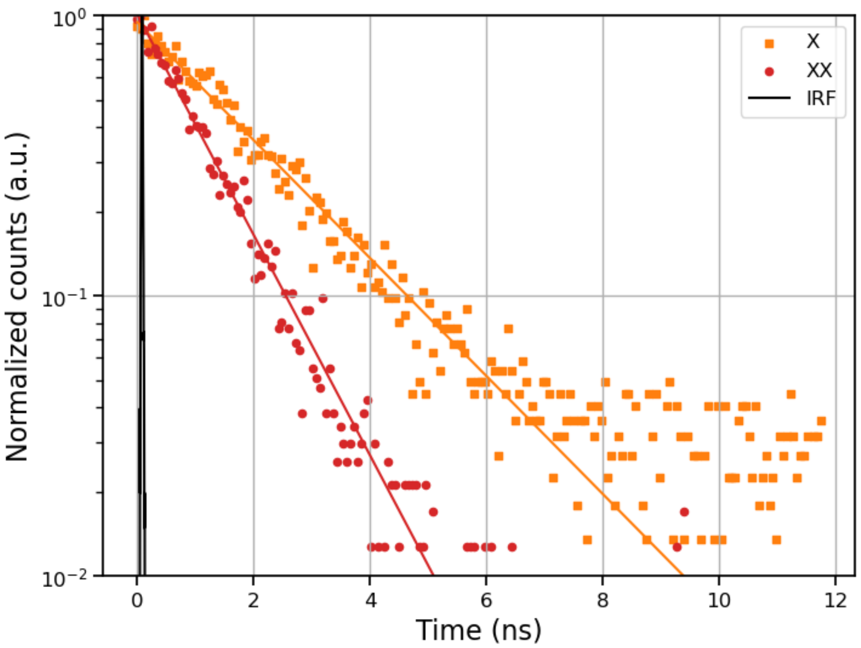

Figure 3: **Lifetime of X and XX under p-shell excitation** The lifetime of X and XX, extracted from single exponential fit is 2.06 ns and 1.1 ns, respectively

as well as the instrument response function (IRF). By fitting the data to a single exponential, we obtain a lifetime of 2.06 ns for X and 1.1 ns for XX. It should be noted that the lifetime extracted from the fitting for the exciton does not directly refer to radiative lifetime of the exciton itself. Because of the cascaded emission process, an exciton photon always comes after biexciton emission. What was measured is actually the time constant of the whole cascaded process. The actual radiative lifetime of the exciton can be extracted from the cross-correlation measurements, which revealed lifetime of 1.61 ns here.

S2. Power dependence of neutral and charged excitonic states

In the main text, we indicated that other radiative paths in nanowires compete with the conventional cascaded emission, which consequently affects the power dependence of the cascaded photon pairs. Here we further show the power series of the studied nanowire

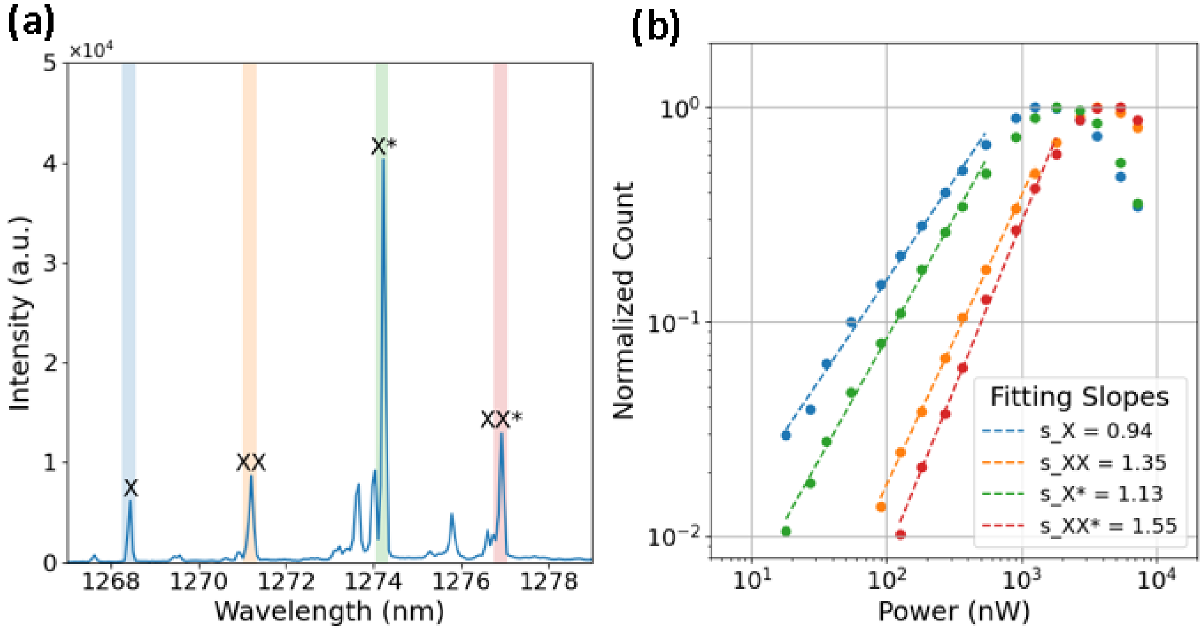

Figure 4: **Power dependence of the investigated nanowire using aboveband excitation** Figure (a) shows the PL spectrum of the nanowire under aboveband excitation. Figure (b) shows the power series corresponding to excitonic emissions.

under aboveband excitation. A linear power relation is observed for the exciton and charged exciton, while nonlinear relations are obtained for both biexciton and charged-biexciton cases. We conclude that in the investigated nanowire system, carriers tend to form and radiate from charged-excitonic states, leading to higher PL intensity from the emission of charged excitons. As discussed in Section S1, changing the excitation scheme affects carrier dynamics in the nanowire, ultimately leading to a different power relation under p-shell excitation.

S3. Single-photon purity of Biexciton and recapturing mechanism

As shown in the main text, the pulsed $g^2(0)$ value calculated from raw data is not as good as expected for the biexciton case. When plotting the data with higher time resolution, there

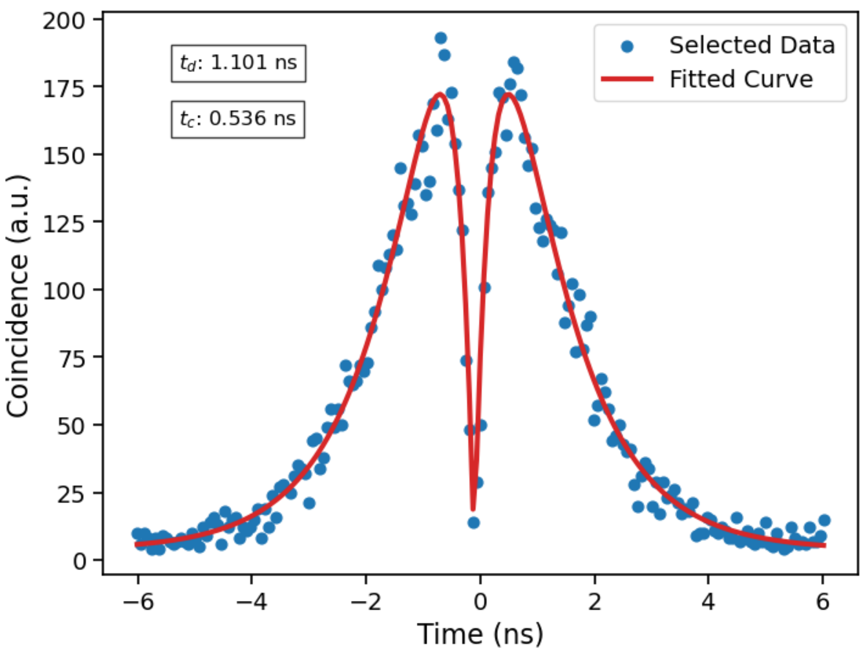

Figure 5: **Auto-correlation measurement of the biexciton.** A zoomed-in view of the bunching peak at zero time delay. The red line is a fit to the model described in the text.

is a clear antibunching dip at zero delay. To explain this phenomenon in detail, we fit the data with the following equation:

$$y(t) = C + A \exp\left(-\frac{|t-t_0|}{t_d}\right)\left(1-\exp\left(-\frac{|t-t_0|}{t_c}\right)\right), \tag{1}$$

where C represents the background level contributed by nearby peaks, and A is the amplitude of a double-sided exponential decay peak. Here we extract the time constants of the radiative process t_d, and the recapturing process, t_c. Due to the nature of the single-photon emitter, the QD should emit only one biexciton photon followed by one exciton photon for each laser pulse, resulting in no coincidence at the zero-delay peak in both the exciton and biexciton cases. However, when we excite the QD at saturation power and provide enough carriers, the QD tends to capture another electron-hole pair to form a biexciton state again, rather than emitting an exciton photon. As a consequence, we observe such recapturing behavior in the biexciton case while maintaining high single-photon purity in the exciton case. It is noteworthy that this effect does not cause any issue for quantum state tomogra-

phy because each measured photon pair is guaranteed to originate from the same cascaded emission process.

S4. Blinking analysis

As shown in Figure 2d and 2e of the main text, the measured second-order autocorrelation functions $g^{(2)}(\tau)$ for both the exciton and biexciton exhibit bunching behavior on long timescales. This slow bunching is indicative of blinking dynamics, where the quantum dot intermittently switches between emissive (on) and non-emissive (off) states due to charge trapping and recapture in its environment.

To quantify this behavior, we used the model described by von Helversen et al.[1]

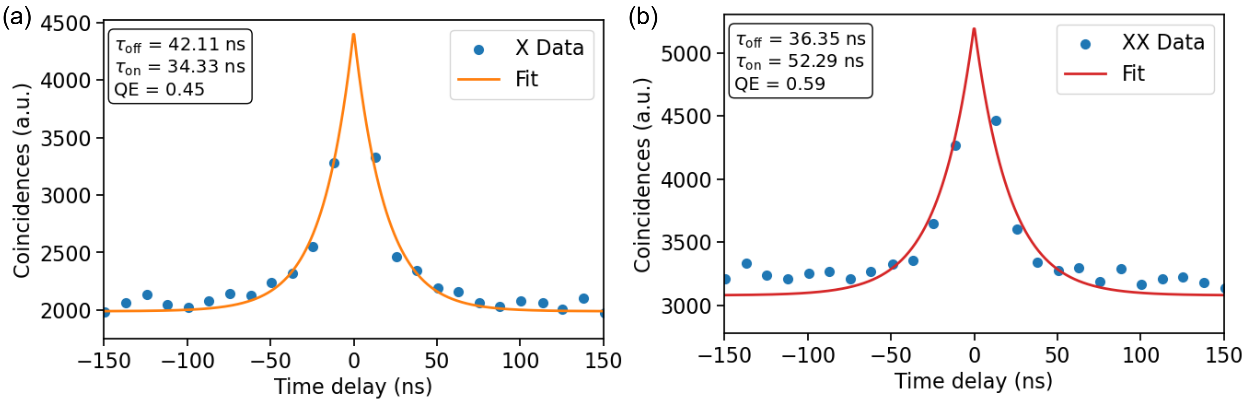

Figure 6: **Blinking behavior of the nanowire QD under p-shell excitation for (a) exciton and (b) biexciton**

Figure 6 shows the measured $g^{(2)}(\tau)$ side peaks for both X and XX under pulsed excitation, together with the corresponding fits using the blinking model. From these fits, we extract the following characteristic blinking timescales:

- **Exciton (X):** $\tau_{\text{on}} \approx 34.33$ ns, $\tau_{\text{off}} \approx 42.11$ ns
- **Biexciton (XX):** $\tau_{\text{on}} \approx 52.29$ ns, $\tau_{\text{off}} \approx 36.35$ ns

These results confirm the presence of blinking behavior under p-shell excitation. Using

these parameters, we estimate the quantum efficiency (QE) of the source — defined as the fraction of time the quantum dot resides in the emissive state using:

$$\mathrm{QE} = \frac{\tau_{\mathrm{on}}}{\tau_{\mathrm{on}} + \tau_{\mathrm{off}}}$$

From this, we obtain a QE ≈ 0.45 for the exciton, and a QE ≈ 0.59 for the biexciton.

While the blinking exhibited here does not preclude generation of polarization-entangled photon pairs with high fidelity, it can reduce the effective brightness and determinism of the source. Mitigation strategies can include white light illumination to passivate charge traps and stabilize the local charge environment, resonant excitation schemes to reduce unwanted carrier generation, and surface or growth optimization. By adopting such mitigation techniques, we can expect the reported 12.5 % source efficiency reported here to be further enhanced.

S5. Source brightness and setup efficiency

The total throughput of the optical setup, including the objective lens, beam splitter, fiber coupling, fiber mating sleeves, and transmission grating, was measured to be 0.8%, with the superconducting nanowire single-photon detector (SNSPD) contributing an additional 50% efficiency. At an excitation repetition rate of 80 MHz, we measured a photon count rate of approximately 40 kcps for both X and XX when excited into the p-shell. Taking into account the combined efficiency of the optical setup and the SNSPDs, the estimated photon rate from the source at the first lens is approximately 16.67 MHz, which corresponds to 12.5% of the 80 MHz excitation repetition rate. This demonstrates the high brightness of the nanowire quantum dot source.

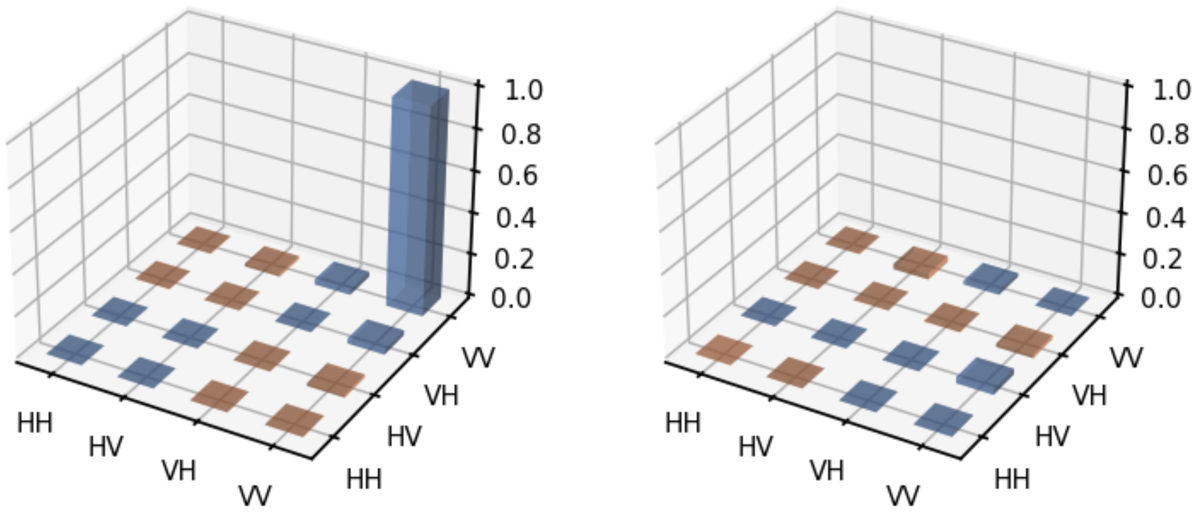

Figure 7: **A calibration state tomography measured using classical light prepared in a vertical polarization state**

S6. Calibration of the tomography setup

To validate the accuracy of the quantum state tomography setup used for measuring entanglement generated by a quantum dot, a calibration measurement was performed following the acquisition of projection angles for 36 polarization bases. A laser source, set to a well-defined VV polarization state using a quarter-wave plate and a half-wave plate, was used as the input to the tomography setup. Quantum state tomography was performed with 16 polarization projections, allowing for the reconstruction of the input polarization state based on the code in Ref.[2] The reconstructed density matrix from the measured projections is shown in Figure 7. We obtain a fidelity of 0.998 ± 0.074 with respect to the ideal VV polarization state, confirming the high accuracy and reliability of the tomography setup for characterizing the entangled state generated by the quantum dot.

S7. Cross-correlation measurements

Here, we present 16 raw cross-correlation measurements of the XX-X cascade, selected from the 36 measured projections that were used to reconstruct the density matrices at different time bins throughout the cascade. The modulation observed in the cascade is a result of the fine-structure splitting in the quantum dot, which leads to a time-evolved entangled state.

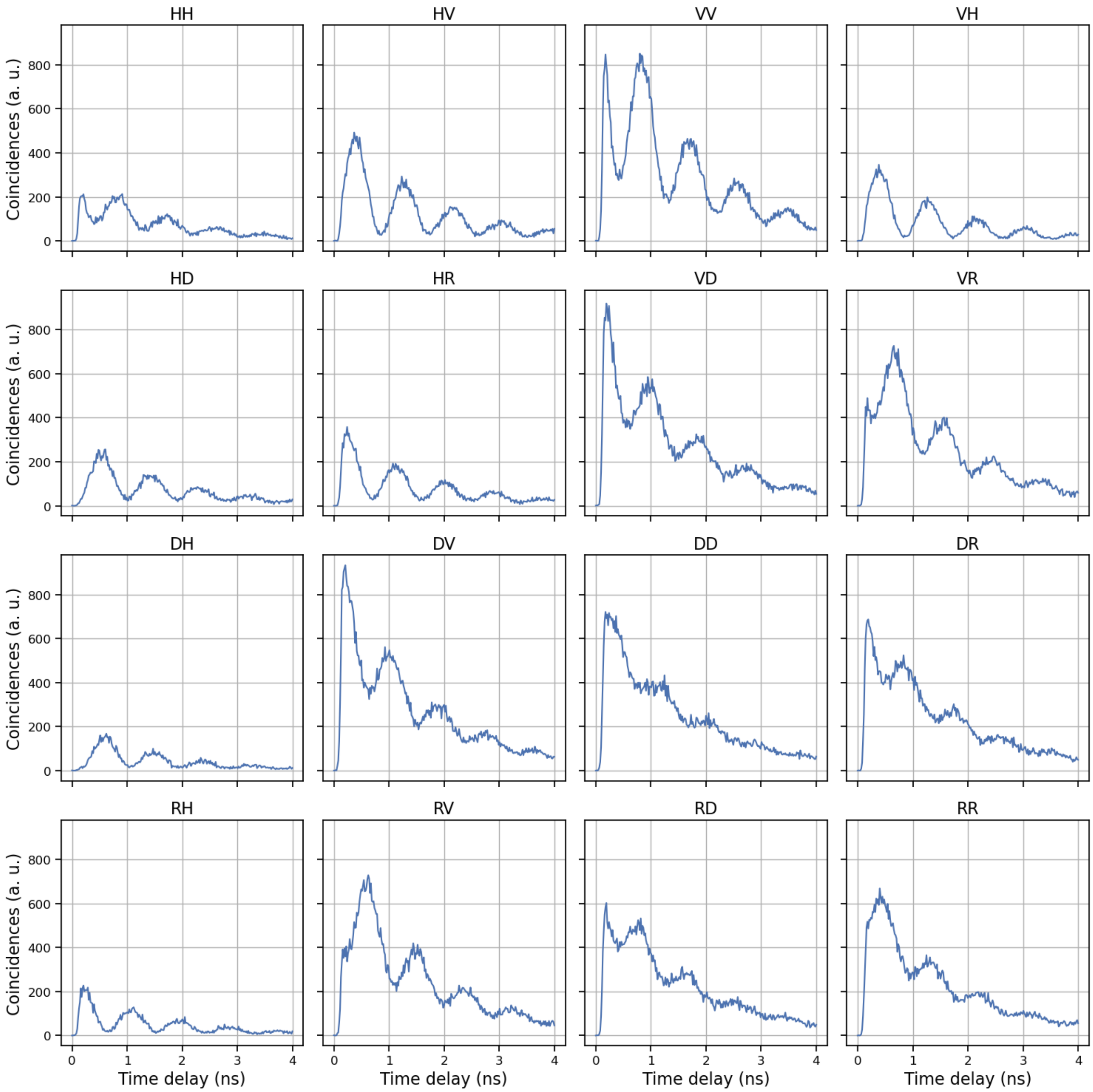

Figure 8: **Raw cross correlation measurements for 16 polarization projections out of the 36 measured projections.**

References

(1) Wei, Y.; Liu, S.; Li, X.; Yu, Y.; Su, X.; Li, S.; Shang, X.; Liu, H.; Hao, H.; Ni, H.; Yu, S.; Niu, Z.; Iles-Smith, J.; Liu, J.; Wang, X. Tailoring solid-state single-photon sources with stimulated emissions. *Nat. Nanotechnol.* **2022**, *17*, 470–476.

(2) Fokkens, T.; Fognini, A.; Zwiller, V. Optical Quantum Tomography Code. `https://github.com/afognini/Tomography`, [Online; accessed 10. Jan. 2025].
